# Field Measurements and Guidelines for the Application of Wireless Sensor Networks to the Environment and Security

**DOI:** 10.3390/s91210309

**Published:** 2009-12-18

**Authors:** Víctor P. Gil Jiménez, Ana García Armada

**Affiliations:** Department Signal Theory and Communications, University Carlos III de Madrid, Av. de la Universidad, 30, 28911 Leganés, Madrid, Spain; E-Mail: agarcia@tsc.uc3m.es

**Keywords:** wireless sensor networks, WSN, transmission problems, micaZ, application design, remote sensing sensors

## Abstract

Frequently, Wireless Sensor Networks (WSN) are designed focusing on applications and omitting transmission problems in these wireless networks. In this paper, we present a measurement campaign that has been carried out using one of the most commonly used WSN platforms, the micaZ from Crossbow^©^. Based on these measurements, some guidelines to deploy a robust and reliable WSN are provided. The results are focused on security and environmental applications but can also be extrapolated to other scenarios. A main conclusion that can be extracted is that, from the transmission point of view, a dense WSN is one of the best choices to overcome many of the transmission problems such as the existence of a transitional region, redundance, forwarding, obstructions or interference with other systems.

## 1. Introduction

Nowadays, we are plunged in a measured world. Most of the physical phenomena are measured for monitoring or security purposes, e.g., temperature at all the stages in a production's chain, or different rooms in an office; pressure and resistance at different stages for specific pieces [1], *etc.* In most of the quality standards in production management such as ISO 28000:2007 [2] or ISO/TS 16949:2002 [3], it is mandatory to monitor every single process to guarantee that the final product fits some quality parameters. Therefore, the quantity and variety of sensors has been growing over the last decade. However, not only there is a need to measure the desired parameters but also it is important to be able to collect and fuse all these measurements in order to evaluate them and, if needed, to act in consequence. This collecting task may be very time-consuming or expensive to be manually accomplished. A physical wire to connect sensors to a central point for collecting data solves the problem for a constrained size or fixed system, but may not be the solution when the elements to be monitored are either wide spread or their positions are subject to change as e.g., in environmental applications such as vineyard [4] or soil monitoring [5]. For these applications, Wireless Sensor Networks (WSN) have been proposed [6] to fit the requirements of monitoring, collecting and processing huge amounts of data from different sources and natures.

Due to the rapid growth of these technologies, flexibility and easiness of deployment, WSN are used for a wide range of applications, which were traditionally based on wired sensor systems. Now, there exist WSN applied to multitude of applications, as for example, environment [4, 5] or security [7].

The number and the nature of applications is very wide, however, not always their design is properly studied or analyzed. For example, in [8], authors exposed that WSN security must be determined by the application and so the design. However, it is important to analyze the WSN application from the point of view of the limitations and transmission characteristics too. In [9], some measurements have been performed in a WSN based on Moteiv [10] (at 2.4 GHz) for industrial applications, where the network is known and a master equipment polls the sensors for data acquisition (*i.e.*, collisions are avoided). In [11], the authors look for a good metric to indicate the quality of the link in a WSN at 900 MHz in indoor office scenarios, selecting the best and the worst channel. In [12], a measurement campaign at 433 MHz in indoor scenarios (parking plenty of cars) is carried out in order to characterize the path-loss with the distance for WSN in this frequency-band. Finally, in [13], an outdoor measurements campaign at 5.8 GHz for the statistical characterization of the propagation in a Durian Orchard forest is presented. In this paper, we present the results of a field measurement campaign that has been carried out to characterize the propagation and the performance behavior (considering distance, height, payload size, obstacles and interference) of one of the most utilized WSN platforms, the MICAz platform from Crossbow [14], which works in the 2.4 GHz. Based on this measurement campaign, some clues and guidelines are provided towards efficient, robust, reliable and successful design of any application, especially focusing on security and environmental uses.

The structure of the paper is the following. After this introduction, a description of Wireless Sensor Networks technology is presented in Section 2., where also the main challenges are outlined. Next, in Section 3., some applications for security and environment are introduced and, in Section 4., the main measurements and guidelines for the correct design of a WSN application are shown. Finally, in Section 5., some conclusions are drawn.

## 2. Wireless Sensor Networks

The wireless sensor networks are the combination of a sensor board, which provides sensing capabilities, and a wireless communication board (usually denoted as mote), that offers the ability to send the measured data by a radio link to a sink or to another node, conforming, this way, a wireless network. Thus, the nodes can be spread covering the area to be monitored and then, data is easily collected via radio by the sink node. This capability is one of the main advantages of these WSN that lends to them an enormous variety of uses. Another advantage is the ad-hoc characteristic, which simplifies the management and maintenance: nodes establish an ad-hoc network that does not require manual configuration and allow self-configuration in case of nodes damage or the entrance of new nodes in the network. Besides, this behavior confers robustness to the WSN. However, this ad-hoc configuration also implies more complexity and challenges from the point of view of higher layer protocols, which are out of the scope of the paper.

### 2.1. Wireless Technology

There exist several standard technologies that allow the development of WSN, namely, IEEE 802.15.4 [15, 16] and ZigBee [17, 18], Z-Wave [19] and other proprietary technologies such as INSTEON [20], the batery-less EnOcean [21] (which make use of energy harvesting), or the open source ONE-NET [22].

The IEEE 802.15.4 [16] defines the two first levels of the Open System Interconection (OSI) layer model, *i.e.*, the physical and the Medium Access Control (MAC) layers, whereas ZigBee [18] specifies the protocols on upper layers, *i.e.*, Network, Transport and Application layers. The other proposals, either use IEEE 802.15.4 as physical layer or a proprietary one. These higher layers provide the above mentioned self-configuring characteristic.

#### IEEE 802.15.4

The standard [15] defines three frequency bands of operation, namely, 868.0–868.6 MHz for Europe (1 channel), 902.0–928.0 MHz for USA (10 channels) and the Industrial, Scientific and Medical (ISM) at 2.4–2.4835 GHz worldwide (16 channels); and its amendment [16] adds three new optional bands, 250–750 MHz, 3.244–4.742 GHz and 5.944–10.234 GHz.

The standard uses Direct Sequence Spread Spectrum (DSSS) [23] for transmitting the signal in the first frequency band and also Ultra Wide Band (UWB) [24] for the three new bands added by the amendment. Two main goals are reached by using DSSS, the first one, the signal does not interfere other systems in the same band (especially important for ISM band), and the second, it is robust against narrow-band interference from other systems. Besides, DSSS allows devices to sleep without the requirement for close synchronization.

On the band 868–868.6 MHz, the modulation is Binary Phase Shift Keying (BPSK) (for 20 kbps), and the optional Offset-Quadrature Phase Shift Keying (O-QPSK) (for 100 kbps) or Amplitude Shift Keying (ASK) (for 250 kbps), whereas for the 902–928 MHz band, the modulation is BPSK (for 40 kbps), and the optional O-QPSK or ASK (for 250 kbps). Next, in the 2400–2483.5 MHz band, only O-QPSK is possible at a rate of 250 kbps. Since equipments in this last band can operate worldwide, most of the WSN have been deployed using the 2.4 GHz technology.

The standard sets the minimum amount of energy needed to transmit to -3 dBm (0′5 mW) and the minimum sensitivity at the receiver is -92 dBm.

#### ZigBee

As mentioned before, ZigBee [17, 18] is the protocol stack for higher layers, commonly used in a WSN. The main tasks defined in the specification are devoted to, namely, Network Formation Procedures (NFP), Mesh Networking and Path Diversity (MNPD), Network-Layer Frequency Agility (NLFA) and End-to-End Acknowledgement and Retransmission (EEAR). Although ZigBee has been designed to complement the IEEE 802.15.4, it could also work with other similar physical layer protocols. ZigBee provides self-organizing, self-healing dynamic mesh network and the possibility of thousand of devices per network, which make it attractive for dense ad-hoc networks.

Since ZigBee has been designed for WSN, a very low duty cycle is used. Besides, static and dynamic star and mesh networks can be managed with low latency. Moreover, it has the ability to make the nodes remain quiescent for long periods without communications.

### 2.2. Sensor Technology

As the variety of magnitudes to be measured is large, so is the number of sensors; however, in this paper we are only focused on those most common for security and environmental applications, such as temperature, light, pressure, smoke, humidity …

The main characteristics for these sensors are the following: they must be able of low-consumption, small and very stable with time and weather (since most of them will operate outdoors during long periods of time without supervision). Usually, technology for these sensors is Micro-Electro-Mechanical Systems (MEMS)-based.

For our testbed, the sensing board MTS310 has been chosen, equipped with a light sensor, a thermistor, a 2-axes accelerometer and a 2-axes magnetometer [25]. The light sensor is a simple CdSe photocell which maximum sensitivity at the light wavelength of 690 nm. With typical on/off resistance, of 2 kΩ/520 kΩ. The thermistor is the Panasonic ERT-J1VR103J. It is configured in a simple voltage divider circuit with a nominal mid-scale reading at 25 °C. It should be noted that only one of them can be used at a time since they share the same Analog to Digital Converter (ADC).

The 2-axes accelerometer (ADXL202JE from Analog Devices) is a MEMS surface micro-machined 2-axis, ±2 g device. It features very low current draw (<1 mA) and it has 10-bit resolution. The sensor can be used for tilt detection, movement, vibration, and/or seismic measurement. The bandwidth is up to 50 Hz, the resolution is 2 mG (0.002 G) Root Mean Square (RMS) and the sensitivity 167 mV/G ± 17 %.

The 2-axes magnetometer circuit (HMC1002 from Honeywell) is a silicon sensor that has a unique bridge resistor coated in a highly sensitive NiFe coating. The NiFe coating causes the bridge resistance of the circuit to change. The bridge is highly sensitive and can even measure the Earth's field and other small magnetic fields.

### 2.3. Challenges

The uses of WSN are very wide, and so are the challenges. Most of the applications need very stable and accurate sensors at the same time that they must be low-consumption and small-size not to disturb too much in the area to be monitored. Moreover, they have a limitation in the voltage requirements due to either the use of small-size batteries or harvesting energy procedures. Besides, the majority of the energy will be used for the wireless communication module.

From the network point of view, on one hand, the motes should be as simple as possible in order to reduce the power-consumption and the size, and, on the other hand, they must be powerful enough to deal with an ad-hoc (even time varying) configuration where, not only physical layer protocols need to be implemented but also higher layers such as routing and multi-hop protocols.

Regarding the communications module, it must be able to transmit using very low-power but at the same time, the operation link should be as large as possible, *i.e.*, the receiver sensitivity should be high and some signal processing is recommended to be able to reduce the interferences due to other devices or systems. As it will be seen later in this paper, WSN in the ISM band are very susceptible to be interfered by other systems in the same band such as Wifi, Bluetooth or microwaves [26, 27, 28].

## 3. WSN Applications for the Environment and Security

The applications of WSN for environmental research are very broad since their ad-hoc nature fits perfectly with the characteristics of the nature. Besides, the number of elements to be measured, sensed or tracked, are large, which configures WSN as a good candidate technology. Application spectrum covers, from the general habitat monitoring in Great Duck Island and the James reserve [29], to more specific targets such as monitoring of birds movements [30] or tracking of small animals (such as toads) [31]. From forest fire or flood detection [32, 33, 34, 35], pollution management [36] or meteorological magnitudes measurement [37], to bio-complexity mapping [38, 39], ecology [40], geophysical supervision [41, 42] or precision agriculture [4, 43, 44], among others.

Regarding security, the WSN provide a very interesting area of application. Many projects and solutions are being proposed such as in [45], where a homeland security network is designed using WSN for perimeter surveillance within project UbiSec&Sens [46], or BODAS, that detects threats against the security and safety of pipelines [47]. Many applications are focused on moving targets as in [48], video surveillance of humans and vehicles in urban areas [49] or traffic surveillance [50].

Due to their ad-hoc property, self-configuration and inherent robustness against attacks, WSN are very interesting for military applications too. We can cite as an example [51], for battlefield surveillance or specific-field applications such as TADAS, which is a tactical sensing system to detect and classify the intruders [47].

Most of the wireless sensor networks above presented have been designed with the application as a main target. In the following section, some measurements to characterize the WSN transmission for MicaZ modules from Crossbow [14] are presented, and some clues and guidelines are extracted from our experience.

## 4. Discussion of Measurement Results

In this section, several measurements carried out using the MicaZ motes from Crossbow [14], equipped with MTS300 and MTS310 sensor boards and an ATmel ATmega 128L microprocessor at 7 MHz [25], are shown to evaluate the different parameters to be had into account when designing a network for a specific scenario or application. The mote wakes up every certain time and obtains data (the temperature, the magnetic field and the acceleration) from sensors, builds the packet and sends it to the sink.

**Figure 1.**
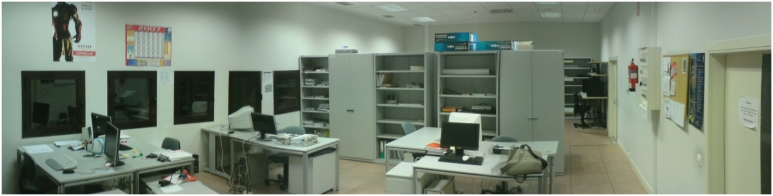
Office scenario picture.

**Figure 2.**
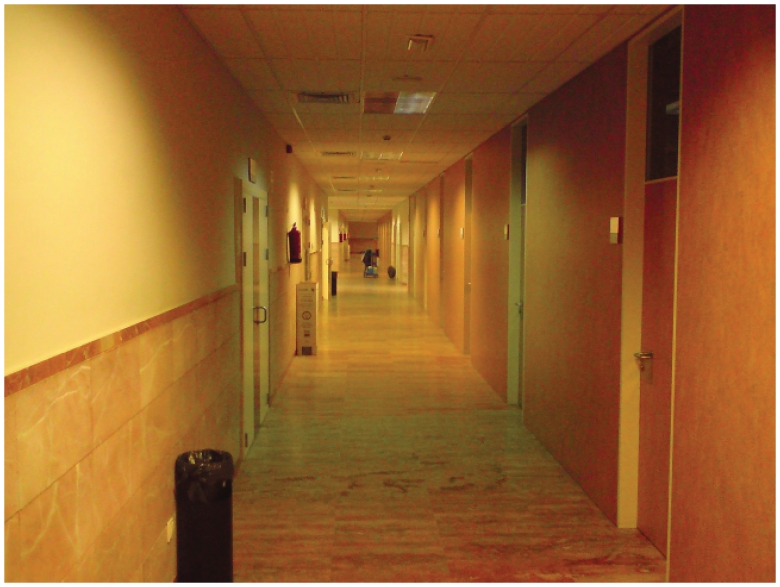
Corridor scenario picture.

Two different scenarios have been tested, both indoor. The first one is a typical office scenario of size 20 × 10 × 3 m, equipped with 8 work-places with computers and shelves (Figure 1). The second is a typical corridor scenario of size 80 × 2 × 3 m (Figure 2).

Since in WSN the data rate available is not very large, usually the data are sent without Forward Error Correcting (FEC) codes in order to avoid unnecessary redundancy and to keep the operations carried out by nodes as simple as possible. Thus, data are sent unprotected and only if the packet is correctly received the network will be able to decode the information, otherwise, a retransmission is needed. For this reason, the Packet Error Rate (PER) is used as a measurement of the network performance. Another quality measurement is the RSSI (Received Signal Strength Indicator) [52] that takes into account the signal received power and is usually utilized by higher layer applications for evaluating the link quality (and act accordingly). We will see that this parameter is not always valid to determine the link quality and it should be carefully used.

In [53], the transmission regions are divided into three, namely, connected, transitional and disconnected, *i.e.*, there is a region where nodes are able to correctly transmit/receive with a low error probability (connected), and then, a region where they are too far away and they are not able to transmit/receive (disconnected). In between, there is the transitional region. Within this region, the received power is near the sensitivity of the receiver and thus, there exists a large variance in the results. Therefore, we must take carefully into account this region to avoid placing sensors in there, if possible, because its reliability is low.

### 4.1. Power Measurements

**Figure 3.**
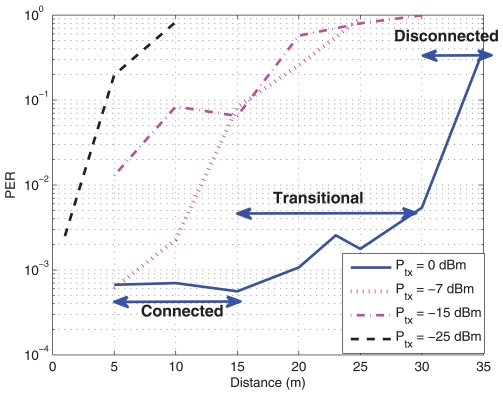

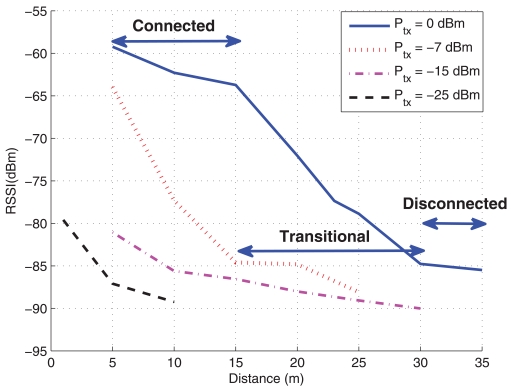
PER and RSSI against distance for different transmission powers. Corridor Scenario. (a) Packet Error Rate (b) Received Signal Strength Indicator

As mentioned before, one of the most limiting parameters in WSN is the power consumption and thus, its use will determine the autonomy (life-time without maintenance) and coverage of the network. In Figure 3a, the average PER against the distance for different transmission powers is shown. 100.000 packets at 50 packets/sec rate have been sent in the corridor scenario at a height of around 50 cm above the ground. It can be seen that, as the transmit power decreases, the PER increases. Especially for transmit values below -15 dBm, the maximum distance should be limited to 10 meters (at 10% PER). It also can be observed that there exists a region around 15 meters where the node experiences the transitional region [53]. Another interesting conclusion from these figures is that there exists a cut-off like distance above which the probability of error experiences a rapid increase, and thus, it can not be used (disconnected). These regions will depend on the transmit power. In Figure 3b, the RSSI is shown and it can be seen that below a RSSI of -83 dBm, there is a large error probability. In these figures the three regions for a transmit power of 0 dBm have been indicated.

**Figure 4.**
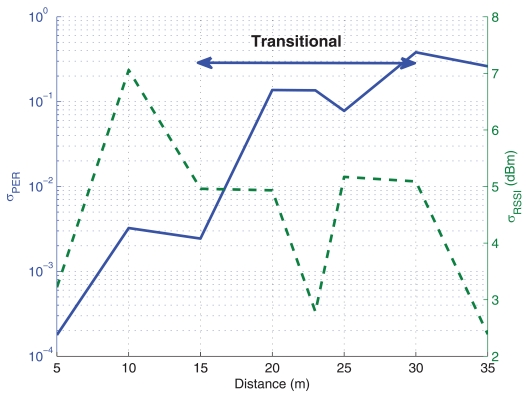
Standard deviation for PER and RSSI. Corridor Scenario.

**Figure 5.**
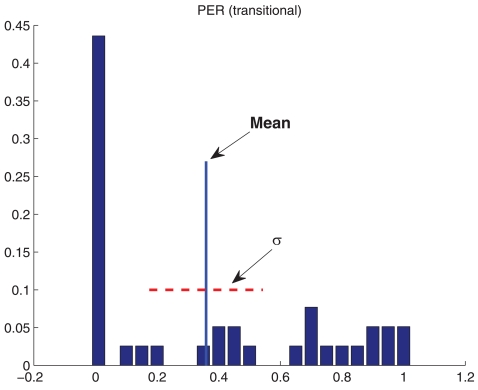

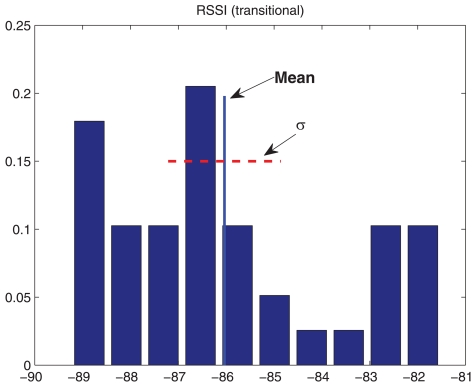
PER and RSSI histogram for the transitional region. Corridor Scenario. (a) Packet Error Rate (b) Received Signal Strength Indicator

In order to statistically characterize the data, experimental measurements have been carried out several times and, in Figure 4, the standard deviation for the obtained data is shown, where the ordinate on the left represents the standard deviation for PER and the ordinate on the right, for the RSSI. It can be seen that, for PER, the standard deviation increases drastically at the transitional region. Indeed, this is one of the characteristics of this region, a large variance in error probability, mainly due to the fact that the receiver is near its sensitivity. Moreover, in Figure 5, the histogram of measurements for a distance of 15 m (transitional region) is shown. It can be observed that, although almost half of the trials obtained low PER, there is a large variation in these data. In the case of RSSI, it can be seen that it is close to uniformly distributed with large variance too. For these reasons, it is not advisable to place motes within this transitional region.

### 4.2. Dependence on the Height above the Ground

**Figure 6.**
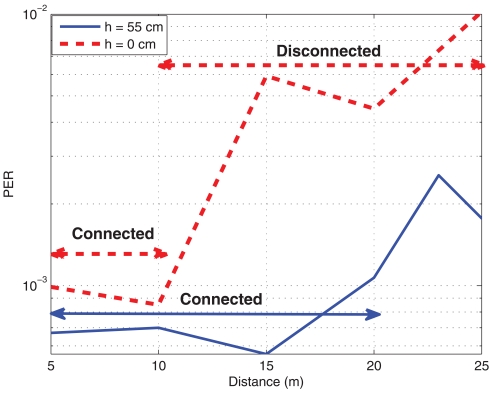

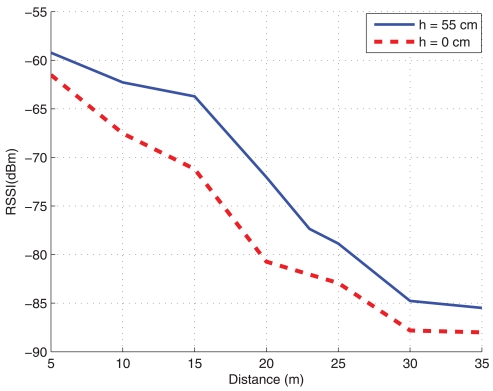
PER and RSSI against distance for two mote heights. Office Scenario. (a) Packet Error Rate (b) Received Signal Strength Indicator

When designing the location of the motes, it is important to know that not only the distance between them is important, but also the height where the nodes are located above the ground. Often, we can not decide where to put the motes and moreover, the position may be time varying. However, if we have the possibility to place motes, it should be remembered that the higher the better. In Figure 6, it can be seen that a mote near the floor will experience a reduction of the size of the connected region and even an on-off behavior, where no transitional region is present. The measures have been taken at maximum transmit power of 0 dBm. The reason for this performance is mainly due to two factors: the radiation pattern of the dipole changes near a ground plane for low elevations, and placing the motes higher it is more likely that they have line of sight transmission. The effect of the modification of radiation pattern will also exist if motes are placed near the ceiling, however, usually the line of sight is guaranteed when placing motes at the ceiling and therefore, the furniture will disturb less this link. Besides, since usually offices have suspended ceiling, the radiation pattern will not be modified anyhow.

### 4.3. Payload Measurements

Another important parameter, usually disregarded in WSN, is the payload size of data. Since motes are simple and with small memory, the packet size becomes important. In fact, this memory must be used to allocate not only the received packet, but also for the operating system and the different software modules to perform the desired tasks in the network, such as the scheduler or the packet forwarder. Since there is the possibility that not all the nodes can reach the sink directly, the packet might be forwarded to several intermediate nodes. On top of that, the probability of incorrect decoding for a large packet is higher.

**Figure 7.**
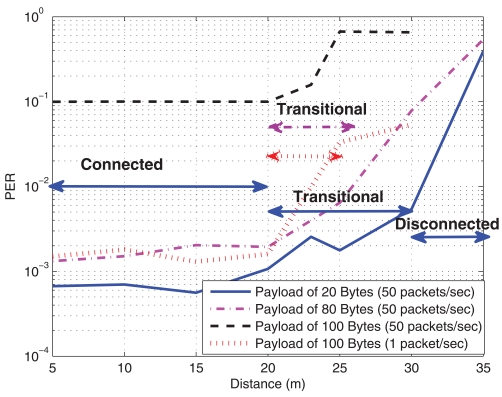

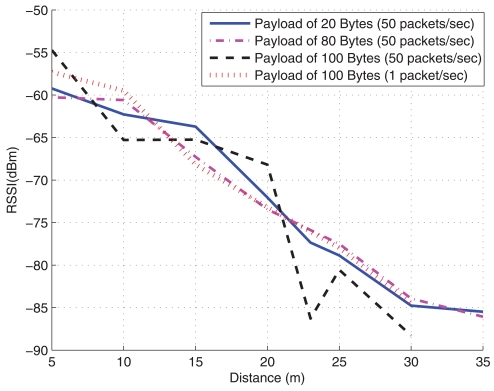
PER and RSSI against distance for different payload sizes. Corridor Scenario. (a) Packet Error Rate (b) Received Signal Strength Indicator

In Figure 7a, the PER is shown for different payload sizes, from 20 Bytes (low data size) to 100 Bytes (large packet), at 50 packets/sec (high load) in a multi-hop scenario, where motes need a forward node to reach the sink. Also a curve with a lower transmission rate of 1 packet/sec is plotted to show the impact on saturation in the forwarder node. The transmit power was again fixed to 0 dBm.

At the transmitter, there is no problem with a large packet, even larger than 100 Bytes, however, for the sink or the forwarder mote, the amount of used memory is larger (it is likely that several packets would be in the queue to be processed), and the mote may be unstable and cause packet loss or even a mote shut down. In Table 1, the used memory at the sending mote and the sink is sketched. Since our motes had only 4 KBytes RAM, packets larger than 80 Bytes will use more than half of the memory (see Table 1). The memory is also used for other parts in the operating system such as the scheduler, and thus, the sink is close to saturation. This situation can be observed in Figure 7a, which is obtained for the sink mote in the corridor scenario: packet sizes above 100 Bytes are too large and cause a large packet loss mainly due to the saturation. This phenomenon is better appreciated in the curve for 100 Bytes at 1 packet/sec rate and in Figure 7b, where it can be observed that the RSSI is similar for all the packet sizes; thus, the PER is due to memory overflow behavior and not to signal strength. When transmission rate is relaxed, *i.e.*, at 1 packet/sec, the saturation effect is alleviated, and therefore, the behavior is similar to smaller payload at higher transmission rate. Besides, this situation is accentuated with the distance and the error probability also increases. An interesting conclusion that can be extracted from Figure 7a is that the payload size does not substantially modify the connected region but only reduces the transitional region, and this means that the disconnected region is increased. Another conclusion is that RSSI can not be used as link quality parameter in this situation, since it does not give relevant information about the actual PER. A careful design on available memory, payload size and packet transmission rate will be needed from this point of view.

### 4.4. Interference

**Figure 8.**
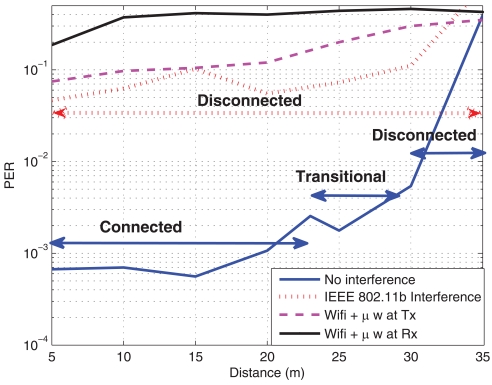

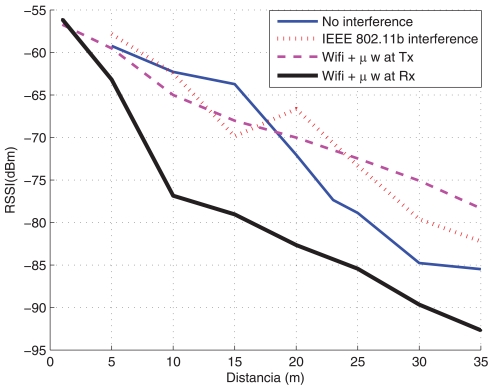
PER and RSSI against distance when IEEE 802.11b interference is present. Office Scenario. (a) Packet Error Rate (b) Received Signal Strength Indicator

As mentioned before, the operation frequency band for our platform is the ISM at 2.4 GHz, the same as IEEE 802.11b/g [54, 55] or microwave ovens. Thus, the effect of their interference should also be taken into account. In order to evaluate this interference, some measurements have been carried out. The Wifi's channel bandwidth is 22 MHz, whereas WSN's bandwidth is 3 MHz. The channel of IEEE 802.11b was fixed to 6 and the channel for the WSN was fixed to 17, which is located just in the middle of the channel 6 for IEEE 802.11b. The Access Point (AP) transmitted at maximum power of 17 dBm, whereas the motes at maximum of 0 dBm. The AP was configured to generate a traffic load of about 30% (mean load). In Figure 8, the PER and the RSSI in the presence of a Wifi access point and a microwave oven are plotted. It can be seen that, when the Wifi is transmitting, the performance of the WSN is very poor and communication can be hardly carried out, *i.e.*, nodes move to the disconnected status. Similar results can be observed when a microwave oven is switched on. It can be noticed that the degradation is more severe when the microwave oven is close to the receiver than to the transmitter. This is because the Signal to Noise plus Interference Ratio (SNIR) is larger when the microwave oven is placed near the transmitter. Another conclusion that can be extracted is that, again, the RSSI parameter can not be used to evaluate the performance in interference environments, since (as it can be seen in Figure 8b) this parameter measures signal strength, and therefore, the interference is considered jointly with the received signal. However, the interference from WiFi can be reasonably mitigated with an adequate frequency channel selection on both, the access point and the WSN. Similar conclusions are extracted in [26, 27, 28], although the characterization of the interference was a laptop instead of an Access Point as in this paper (*i.e.*, less power). As an observation, the channel 26 of IEEE 802.15.4 is not overlapped with no IEEE 802.11b/g, and so, it can be used without interference from these systems, although not from bluetooth or microwaves.

### 4.5. Obstructed Transmission

**Figure 9.**
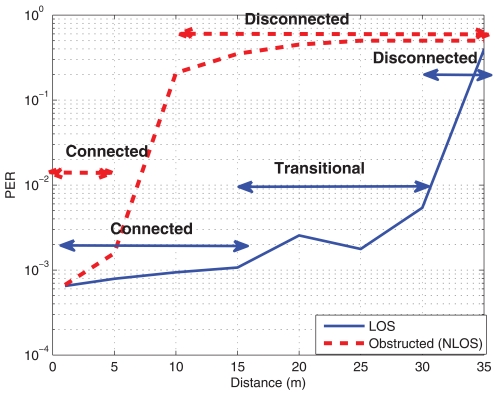

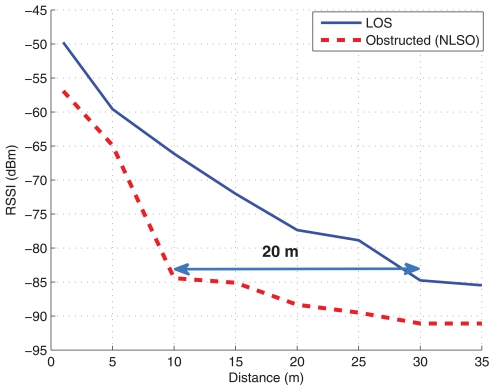
PER and RSSI against distance when nodes are obstructed (inside a drawer). Corridor Scenario. (a) Packet Error Rate (b) Received Signal Strength Indicator

In this section, the performance of the WSN when motes transmissions are severely obstructed by the surrounding objects is evaluated. The transmitter and the receiver were put inside a metallic drawer and, in Figure 9, the PER and the RSSI are plotted. As it can be expected, when covering and obstructing the motes, the connected region reduces and the transitional region almost disappears, increasing the disconnected area. Indeed, it is interesting to note that if a mote is obstructed with a metallic structure, the performance will be reduced to a few meters, mainly due to the Faraday effect (metallic structure shields the electromagnetic waves).

### 4.6. Multi-Hop Transmission

**Figure 10.**
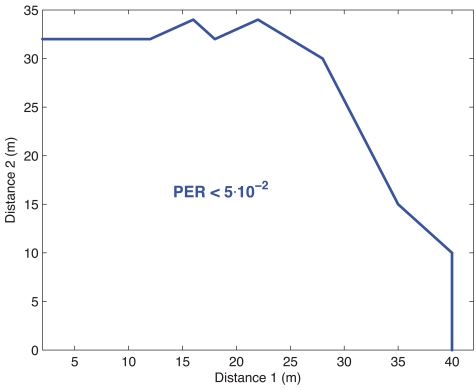
Distance region where two-hop PER is below 5 × 10^−2^. Corridor Scenario.

Finally, in order to evaluate the multi-hop capabilities of WSN, another two-hop measurement campaign has been performed in scenario B. The sending mote was at distance *d*_2_ from relay mote, and this relay mote was at distance *d*_1_ from sink. In Figure 10, the region where the PER was lower than 5 × 10^−2^ is depicted. The transmit power was fixed to 0 dBm at 50 packets/sec. It can be observed that below 30 m, the target PER can be obtained. Besides, the link between the sending mote and the relay is more restrictive than the other due to the double reception (and decoding) process.

### 4.7. Some Guidelines for the Design of WSN

In the following, we outline some guidelines for the design of a robust and reliable WSN. One of the main recommendations is the utilization of a dense network to solve problems such as transitional region, redundance, forwarding, obstructions and interference with other systems.

In a dense WSN, since there is a large number of nodes that are closely located, it is likely that there will be no nodes in the unstable transitional region and the performance will be better, at the expense of more complexity in higher layers and the increase in the probability of collisions. However, in synchronous transmission, a careful design of the *transmission period* for each mote to avoid collisions (that can be done automatically at the sink by using some scheduling algorithm) can significantly reduce the collisions.

Another suggestion is to try not to place motes close to the floor, and, always when possible, keeping line of sight with other nodes. If mandatory, place level-line the motes but also place much higher intermediate forwarders to support the transmission.

In a multi-hop WSN, the packet size becomes important, due to the queues at the intermediate (forwarding) nodes. This situation might place forwarder motes into a near memory overflow and that makes the performance drop dramatically. Since usually applications are designed minimizing the number of transmissions (power consumption criterion), there exist an *acquisition/probe period* and a *transmission period*. During the *probe period*, the node wakes up, takes measurements and stores them into memory. During the *transmission period*, the mote builds the packet with all the measures and sends it (synchronous transmission). This way, the time resolution can be large without increasing transmission frequency. Only asynchronous transmission exists if alarms are configured (if a measured quantity is above or below certain threshold). There is a trade-off however between the *probe period* and the *transmission period* because of the memory constraints and the above mentioned behavior with large packets. A solution may be to divide the large packet into several smaller packets and transmit them in a row, but forward them by different nodes. This way, we obtain a twofold effect, which adds up in a performance improvement, first, we reduce the packet load at each forwarding node, and second, we reduce the PER since packet is shorter. It should be noted that this functionality must be implemented in multi-hop WSN to improve the performance (since it is not provided by default).

Another important consideration is to avoid placing the sink or forwarders near microwave ovens, WiFi nodes or, in general, sources of interference that use the same frequency band. Again, a solution is a dense (and inherently redundant) WSN to reduce the degradation due to interference. Since WiFi or bluetooth nodes can be configured to use a specific frequency channel, as mentioned before, a careful frequency allocation design among access points, bluetooth and WSN will reduce the interference. This is not the case with microwaves.

As seen in this section, the obstruction of motes transmission causes a significant degradation and reduction of coverage. A smart design of the network (for example, placing antennas to avoid obstacles, introduce some forwarder nodes in highly visible points to support transmission from likely obstructed motes) jointly with a dense deployment will give us the better performance.

## 5. Conclusions

WSN are very suitable for environmental or security applications. We have presented and discussed some measurements regarding the transmission performance of WSN focusing on these types of applications. Although most of the applications are designed from the point of view of the security or application, it has been shown that it has to be also designed taking into account the transmission characteristics of these WSN. A careful design on the location (if possible), frequency channel allocation, payload size or strategy, introduce some forwarder nodes to support transmission of obstructed nodes will improve the WSN performance. We can conclude that large and dense WSN are more suitable to reduce the negative effects of interference, transitional region and power limitations of the motes, at the expense of increased complexity in higher layers.

## Figures and Tables

**Table 1.** Used Memory.

	**SendingMote (Bytes)**		**BaseStation (Bytes)**	
**PSDU (Bytes)**	**RAM**	**ROM**	**RAM**	**ROM**
20	262	11398	1184	14914
40	302	11410	1724	14914
80	382	11470	2804	14996
100	422	11470	3344	14996
